# Hepatic Branch Vagotomy Can Suppress Liver Regeneration in Partially Hepatectomized Rats

**DOI:** 10.1155/1993/59691

**Published:** 1993

**Authors:** Masahiro Ohtake, Takeo Sakaguchi, Keisuke Yoshida, Terukazu Muto

**Affiliations:** 1 First Department of Surgery Niigata University School of Medicine Niigata 951 Japan; 2 First Department of Physiology Niigata University School of Medicine Niigata 951 Japan

## Abstract

The role of the vagus nerve in liver regeneration after partial hepatectomy was studied by comparing the effects of hepatic branch vagotomy with those of hepatic branch sympathectomy in rats. The liver weight as a percentage of body weight decreased significantly 7 days after vagotomy compared with the controls and this was associated with a reduction in food intake. There was no difference in the liver weights between the control rats and the pair-fed vagotomized rats. Hepatic sympathectomy had no significant effect on the liver weight. The serum scores indicating hepatic function showed no difference between the control and the vagotomized rats except alkaline phosphatase. The concentration of insulin was unchanged. The number of mitotic hepatocytes remained high at 7 days after vagotomy: These observations led us to conclude that the vagus nerve stimulates liver regeneration, and its effect depends on vagal factors directly and specifically.


*HPB Surgery*, 1993, Vol. 6, pp. 277–286
Reprints available directly from the publisher
Photocopying permitted by license only

© 1993 Harwood Academic Publishers GmbH
Printed in the United States of America

### HEPATIC BRANCH VAGOTOMY CAN SUPPRESS LIVER REGENERATION IN PARTIALLY HEPATECTOMIZED RATS

MASAHIRO OHTAKE, TAKEO SAKAGUCHI*, KEISUKE YOSHIDA and TERUKAZU MUTO

*First Department of Surgery, First Department of Physiology*, Niigata University School of Medicine, Niigata 951, Japan*

*(Received 5 December 1990)*

The role of the vagus nerve in liver regeneration after partial hepatectomy was studied by comparing the effects of hepatic branch vagotomy with those of hepatic branch sympathectomy in rats. The liver weight as a percentage of body weight decreased significantly 7 days after vagotomy compared with the controls and this was associated with a reduction in food intake. There was no difference in the liver weights between the control rats and the pair-fed vagotomized rats. Hepatic sympathectomy had no significant effect on the liver weight. The serum scores indicating hepatic function showed no difference between the control and the vagotomized rats except alkaline phosphatase. The concentration of insulin was unchanged. The number of mitotic hepatocytes remained high at 7 days after vagotomy. These observations led us to conclude that the vagus nerve stimulates liver regeneration, and its effect depends on vagal factors directly and specifically.

KEY WORDS: Vagotomy, sympathectomy, liver regeneration, food intake, water intake, rat

### INTRODUCTION

Studies of neural factors regulating liver regeneration have reported that incorporation of labeled thymidine into liver DNA 24 h after partial hepatectomy was significantly reduced in rats subjected to cervical vagotomy[1], and that not only DNA synthesis and cell proliferation but also restoration of the liver weight were suppressed by subdiaphragmatic vagotomy[2]. These findings have strongly suggested the participation of the vagus nerve in the control of liver regeneration. However, the effect of vagotomy performed at these levels on liver regeneration could be interpreted as resulting from severe systemic side-effects[3] as the vagus nerve innervates many other organs in the abdominal cavity, and its specificity has been controversial. Recently, anatomical pathways of autonomic nerves innervating the liver were determined at branch level, and selective sectioning of the hepatic branch of the vagus nerve in rats has become feasible[4–6]. Furthermore, behavioral studies have demonstrated that hepatic branch vagotomy decreased the consumption of food[4] and water[5] in rats.

Address correspondence to: Masahiro Ohtake, M.D., First Department of Surgery, Niigata University School of Medicine, Niigata 951, Japan.

On the other hand, the regenerative ability of remnant liver has been shown to be subject to the influence of nutritional factors. A period of starvation[7] or protein deprivation[8] suppresses the proliferative response during regeneration.

This study was designed to investigate whether hepatic branch vagotomy can suppress liver regeneration in relation to the nutritional condition.

### MATERIALS AND METHODS

Fifty-four male Wistar rats were used in this study. The animals were reared individually and allowed free access to laboratory chow (MF, Oriental Yeast Co., Osaka) and tap water throughout this experiment. The room temperature was controlled at 24 ± 2 °C with 12 h/12 h light/dark cycles (lighting from 08.00 – 20.00 h). The individual body weight, and food and water intakes were recorded daily between 10.00 – 12.00 h.

When the animals grew to weight about 180 g, partial hepatectomy was carried out under ether anesthesia by the method of Higgins and Anderson[9] between 10.00 – 13.00 h to avoid the effect of diurnal variations[10]. In brief, the median and left lateral lobes of the liver, constituting two-thirds of the total liver mass, were removed. Immediately after hepatectomy, the animals were divided randomly into three surgical groups; hepatic branch vagotomized, hepatic branch sympathectomized and denervated control groups. Each group consisted of six animals. Sympathetic denervation of the liver was performed by resecting the hepatic branch of the splanchnic nerves around the proper hepatic artery with the help of a dissecting microscope[11], while parasympathetic denervation was achieved by selective section of the hepatic branch of the vagus nerve branching off from the left main vagal trunk[4–6] (Figure 1). In the controls the hepatic vagus branch and the hepatic sympathetic branch were merely exposed. The abdominal wall was closed in layers. After surgery, they were returned to their cages and allowed access to food and water immediately.

The animals were anesthetized by intraperitoneal injection of pentobarbital sodium (40 mg/kg) at 10.00 o'clock on 0, 3, 7 and 14 days after the operations. Blood samples for chemical analysis were collected from the tail vein, and the animals were sacrificed to measure the remnant liver weight. To compare the rate of liver regeneration among the groups, the liver weight expressed as a percentage of body weight (relative liver weight) was used as an index. It was preliminarily confirmed that there is a good correlation between the wet and dry liver weights. The specimens from the caudate lobe of the liver were prepared with hematoxylin and eosin, and the proportion of hepatocytes in mitosis was expressed as a mitotic index.

The blood samples obtained were cooled immediately with ice and centrifuged at 2,200 rpm for 20 min. Then the serum separated was stored at –20 °C until measurment of the following parameters of hepatic and renal function, an autoanalyzer (Hitachi-736, Hitachi Co., Tokyo) was used; total protein (TP, Biuret method), albumin (Bromcresol green method), glutamic oxaloacetic transaminase (SGOT, Ultraviolet method), glutamic pyruvic transaminase (SGPT, Ultraviolet method), lactate dehydrogenase (LDH, Wroblewski-LaDue method), alkaline phosphatase (Bessey-Lowry method), total bilirubin (TB, Azobilirubin method), total cholesterol (Tcho, Cholesterol oxidase colorimetric method), blood urea

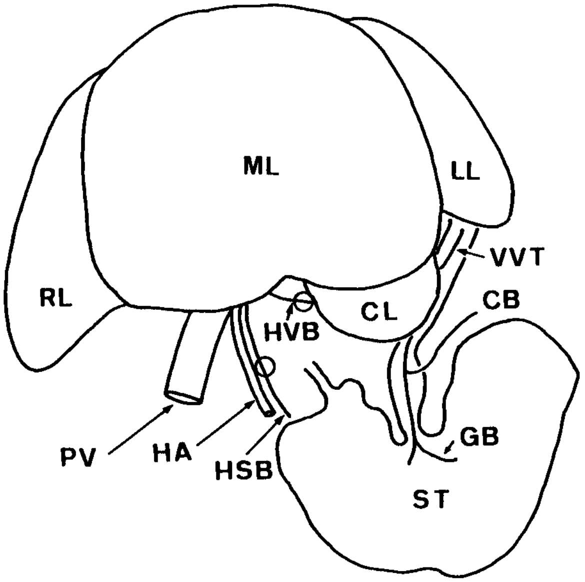

**Figure 1** Schematic diagram of abdominal autonomic pathways below the diaphragm in the rat. Broken circles represent sectioned sites. Abbreviations: CB, celiac vagus branch; CL, caudate lobe of the liver; GB, gastric vagus branch; HA, proper hepatic artery; HSB, hepatic sympathetic branch; HVB, hepatic vagus branch; LL, left lateral lobe of the liver; ML, median lobe of the liver; PV, the portal vein; RL, right lateral lobe of the liver; ST, the stomach; VVT, the ventral vagus trunk.

nitrogen (BUN, Urease ultraviolet method), and creatinine (Cre, Jaffe method). Plasma concentrations of insulin and glucose were determined by double-antibody radioimmunoassay and glucose oxidase method, respectively.

To eliminate the effect on liver regeneration of changes in the amounts of food and water intake due to hepatic vagotomy, a pair-fed group of vagotomized rats was also examined.

The statistical significance of the differences among the results was evaluated by ANOVA and Duncan's multiple range test: $p<0.05$ is defined as significant between the values.

### RESULTS

The effect of hepatic branch vagotomy on liver regeneration after partial (65%) hepatectomy is shown in terms of the liver weight expressed as a percentage of the body weight (Figure 2). The relative liver weight showed a similar pattern of change in the hepatic vagotomized rats and the controls, but comparison of the relative liver weight 7 days after surgery between the groups showed a significant difference in the value and the weight was lower in the vagotomized rats than in the controls ($p<0.05$, $n=6$). However, this difference disappeared 14 days after surgery. ANOVA revealed the group and time differences of $F(1,30)=3.995$, $p<0.05$, and $F(2,30)=11.15$, $p<0.01$, respectively.

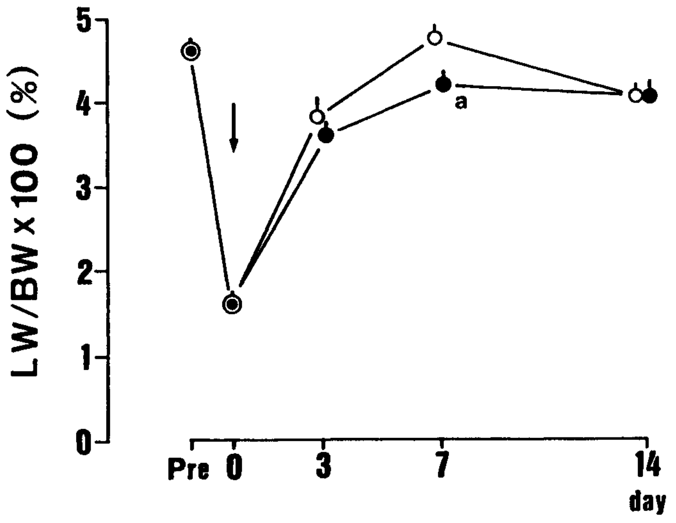

**Figure 2** Changes in liver weight as a percentage of body weight (LW/BW × 100) after hepatic branch vagotomy. The vagotomized control (○) and hepatic branch vagotomized rats (●) are shown. An arrow indicates the time of vagotomy. There are 6 animals in each group, and values are the mean ± SEM. Pre; immediately before the hepatectomy. a, $p<0.05$ compared to the values in the controls.

Changes in food and water intakes in the hepatic branch vagotomized and the control groups were also examined (Figure 3). The total water intake (Mean ± SEM) during the first 7 days after the denervation was 142.7 ± 9.8 ($n=6$) ml in the controls, while it was 154.3 ± 16.9 ($n=6$) ml in the vagotomized rats, indicating no significant difference between the two values. Hepatic branch vagotomy did not alter the daily amount of water intake. ANOVA showed the group and time differences of $F(1,90)=2.466, p>0.05$, and $F(8,90)=3.280, p<0.01$, respectively. However, the total food intake (Mean ± SEM) during the first 7 days after the treatments was 109.7 ± 6.1 ($n=6$) g in the controls, but was 82.7 ± 5.9 ($n=6$) g in the vagotomized group, showing significant reduction of the value in the latter compared to the former ($p<0.05$). Moreover, from the record of food intake, the daily food intake in the control group required about 4 days to recover the preoperative level, but the vagotomized animals needed another day. The daily food intake was reduced significantly in the vagotomized rats compared to the controls almost every day. ANOVA revealed the group and time differences of $F(1,90)=42.47, p<0.01$, and $F(8,90)=39.54, p<0.01$, respectively.

The liver weight expressed as a percentage of body weight and serum scores indicating hepatic and renal function at 7 days after surgery in the hepatic branch sympathectomized, the hepatic branch vagotomized and the pair-fed of the vagotomized rats and the controls are shown in Table 1, together with those obtained from intact animals. On comparing the relative liver weights, there was no difference between the controls and the sympathectomized or the pair-fed groups, but the value in only the vagotomized group was significantly lower than that in the controls ($p<0.05$). ANOVA showed the difference of $F(4,25)=2.684, p<0.05$.

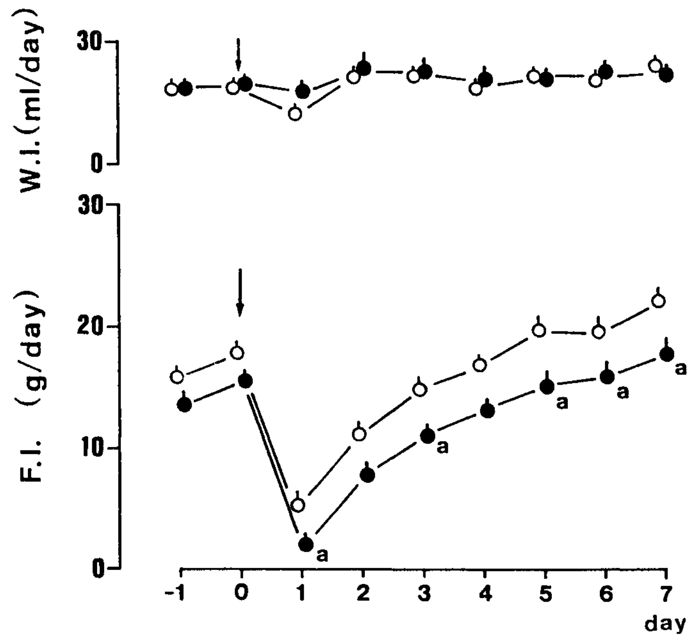

**Figure 3** Effects of hepatic branch vagotomy on water (W.I.) and food (F.I.) intake in partially hepatectomized rats. The vagotomized control (○) and hepatic vagotomized (●) rats were compared. Arrows indicate the time of vagotomy. There are 6 rats in each group, and values are the mean ± SEM. a, $p<0.05$ compared to the values in the controls.

**Table 1** The liver weight as a percentage of body weight (LW/BW × 100) and plasma scores following hepatic branch sympathectomy (HSx), hepatic branch vagotomy (HVx) and pair-fed for vagotomy (PFHVx)

| | *Before Hepatectomy* | *Controls* | *HSx* | *HVx* | *PFHVx* |
|---|---|---|---|---|---|
| LW/BW × 100 (%) | 4.63±0.09 | 4.77±0.12 | 4.64±0.11 | 4.22±0.13[a] | 4.51±0.17 |
| Body weight (g) | 174.3±1.9[a] | 216.3±5.7 | 203.8±5.8 | 202.8±7.4 | 200.2±3.8 |
| SGOT (U) | 68±3 | 84±4 | 101±13 | 77±3 | 108±9[a] |
| Alp (U/l) | 741±26 | 873±43 | 826±42 | 730±14[a] | 794±54 |

Values were obtained 7 days after partial hepatectomy. There are 6 samples in each groups, and values are the mean±SEM. Abbreviations: Alp, alkaline phosphatase. a, p<0.05 compared to the values in the controls.

On comparing the serum scores, the level of SGOT in the pair-fed rats was significantly higher than that in the control ($p<0.05$). The difference by ANOVA was $F(4,25)=4.694$, $p<0.01$. Furthermore, the value of alkaline phosphatase in the vagotomized group was significantly lower than that in the controls ($p<0.05$). ANOVA showed a difference of $F(4,25)=2.389$, $p<0.05$. The hepatic and renal scores, TP, SGPT, LDH, TB, Tcho, BUN and Cre, indicated no significant differences in comparison with those in the controls (data not shown).

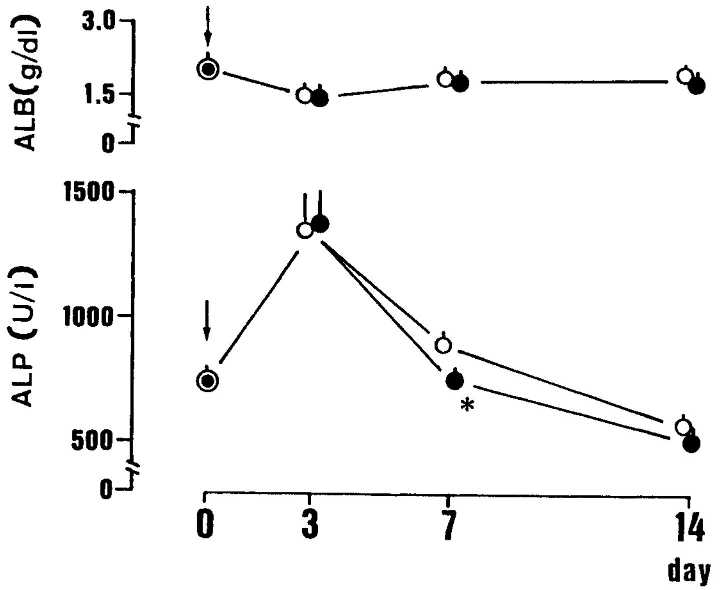

**Figure 4** Plasma concentrations of albumin (ALB) and alkaline phosphatase (ALP) after partial hepatectomy. The vagotomized control (○) and hepatic vagotomized (●) rats were compared. Arrows indicate the time of vagotomy. There are 6 animals in each group, and values are the mean ± SEM. * $p<0.05$ compared to the values in the controls.

Figure 4 shows the changes in serum alkaline phosphatase level after hepatic branch vagotomy together with those in serum albumin and SGPT levels. The values of albumin and SGPT showed no significant differences between the control and hepatic branch vagotomized groups. But the value of alkaline phosphatase 7 days after surgery remained high in the controls, while it returned to the preoperative level in the hepatic vagotomized group, indicating a significant difference ($p<0.05$). ANOVA revealed the group and time differences of $F(1,30)=3.303$, $p>0.05$, and $F(2,30)=52.50$, $p<0.01$, respectively in albumin, $F(1,30)=0.3601$, $p>0.05$, and $F(2,30)=7.576$, $p<0.01$, respectively in SGPT, and $F(1,30)=4.106$, $p<0.05$, and $F(2,30)=37.48$, $p<0.01$, respectively in alkaline phosphatase.

The serum concentrations of insulin and glucose obtained 7 days after the operation from the control, the hepatic branch vagotomized and the hepatic branch sympathectomized rats are shown in Figure 5 in comparison with those in intact animals. Neither insulin nor glucose levels revealed significant differences among the hepatic vagotomized, hepatic sympathectomized and control groups. ANOVA showed the difference in insulin and glucose of $F(3,20)=0.1616$, $p>0.05$, and $F(3,20)=0.6845$, $p>0.05$, respectively.

Figure 6 illustrates the changes in the number of mitotic hepatocytes per 1,000 cells obtained from the remnant liver. The number of mitotic cells in the vagotomized group increased at 3 days after surgery compared with the number before the operation, but was significantly lower than that in the controls ($p<0.05$). But the value at 7 days after surgery stayed the same as that at 3 days after surgery, while the number of mitotic cells in the controls returned to the preoperative level.

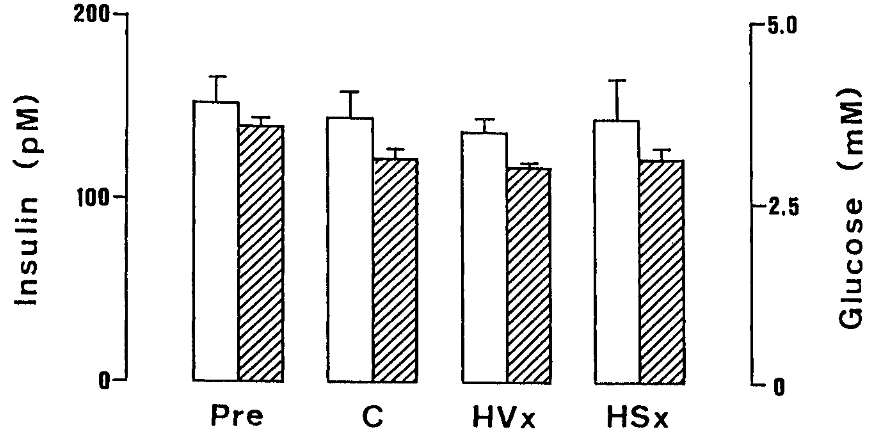

**Figure 5** Plasma concentrations of insulin (open bar) and glucose (hatched bar) 7 days after control vagotomy (C), hepatic branch vagotomy (HVx) and hepatic branch sympathectomy (HSx) in partially hepatectomized rats.There are 6 samples in each group, and values are the mean ± SEM. Pre; immediately before the hepatectomy.

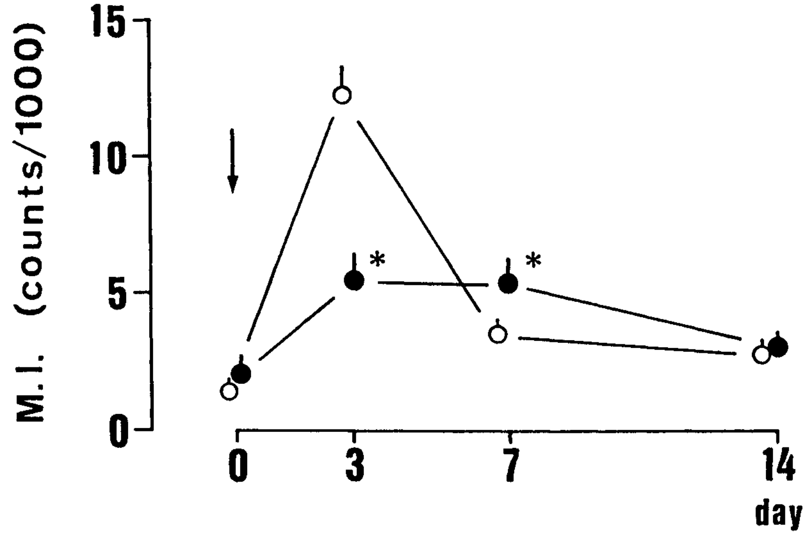

**Figure 6** Alterations in mitotic index (M.I.) of the hepatocytes obtained from the remnant liver after partial hepatectomy. The vagotomized control (O) and hepatic vagotomized (●) rats were compared. An arrow indicates the time of vagotomy. There are 6 samples in each group, and values are the mean ± SEM. * $p<0.05$ compared to the values in the controls.

ANOVA revealed the group and time differences of $F(1,47)=4.501, p<0.05$, and $F(3,47)=70.60, p<0.01$, respectively.

### DISCUSSION

The present study showed that liver regeneration after partial hepatectomy was inhibited by vagotomy conducted at the hepatic branch level (Figure 2). This finding indicates that suppression of liver regeneration by vagotomy is attributed to the direct effect of the nerve, although it can also be interpreted as the result of systemic side-effects, because the vagus nerve innervates not only the liver but also many other organs[3]. Moreover, this result supports previous reports which have showed that liver regeneration was suppressed by vagotomy conducted at the cervical[1] or subdiaphragmatic level[2].

The effect of denervation at the cervical or subdiaphragmatic level was observed with a significant difference at 3 days postoperatively, however, in this study denervation at the hepatic branch level produced its effect at 7 days postoperatively (Figure 2). The time lag in appearance of the effect of denervation may be due to the functional loss of other vagal branches rather than the hepatic branch, in the case of cervical or subdiaphragmatic vagotomy.

It was reported that hepatic branch vagotomy suppressed the eating and drinking behavior of nonhepatectomized intact rats[4,5], which was interpreted as resulting from section of glucose fibers[12] or osmotic fibers[13] in the nerve. However, hepatic branch vagotomy in the partially hepatectomized rats decreased the food intake but not the water intake (Figure 3). Although both glucose and osmotic fibers controlling food and water intakes are transmitted via the common hepatic vagal branch, it is thought that their sites of action in the liver and portal area might be different.

Stirling *et al.*[7] reported that cell proliferation after partial hepatectomy was suppressed in rats deprived of food before or after operation, compared with animals not so deprived. McGowan *et al.*[8] investigated the effects of different diets on liver regeneration, and noted that the animals maintained on a protein-free diet for 3 days have a delayed time course of hepatic DNA synthesis during liver regeneration. Therefore, liver regeneration depends on both diet and food intake. The possibility cannot be denied that the reduced relative liver weight obtained in the hepatic vagotomized animals in this study might be due to reduction of food intake (Figures 2, 3). However, there was no significant difference in the relative liver weights between the pair-fed group and the controls (Table 1), implying that the inhibitory response in liver regeneration observed in this experiment was not due to the reduced amount of food intake but to the loss of a specific effect of the vagus nerve on regeneration. On the other hand, hepatic branch sympathectomy proved to have no influence on liver regeneration, supporting the view of Kato and Shimazu[2] that bilateral major splanchnicectomy at the subdiaphragmatic level produced no effect on regeneration.

With respect to blood chemistry, albumin concentration was unaffected by hepatic branch vagotomy (Figure 4). This suggests that there was no noticeable change in the nutrition of animals when vagotomy produced a reduction of food intake (Figure 3). Moreover, because albumin has been shown to be one of the proteins specifically produced in the liver, our results also imply that the vagus nerve has no role in the production of protein in the remnant liver. The SGOT activity in the pair-fed group of the hepatic branch vagotomized animals was significantly higher than that in the controls (Table 1). GOT is a very common enzyme distributed in erythrocytes, myocardium and many other organs, and has no significant specificity for the liver.

Of great interest is the low value of alkaline phosphatase in the vagotomized group on the seventh day compared to that in the controls (Table 1 and Figure 4). Reilly *et al.*[14] observed histologically that cholinergic nerves innervate bile ducts running along the hepatic vasculature. Moreover, electrical stimulation of the vagus nerve induced an increase in intracholedochal pressure[15], suggesting a direct relationship between the vagus nerve and bile secretion. In general, the serum level of alkaline phosphatase in liver diseases is elevated by bile stasis[16]. Therfore, the elevated activity of alkaline phosphatase could be interpreted as resulting from the bile stasis caused by intracholedochal pressure increase due to vagal stimulation. However, more detailed studies on the exact relationship between the vagus nerve and bile secretion are needed.

Insulin is regarded to be one of the hepatotrophic factors[17], because it exerts direct effects on organelles and stimulates DNA and protein syntheses[18], also its secretion is known to be regulated through the hepatic vagus branch[19]. As insulin secreted from the pancreas is mostly released into the portal vein to reach the liver, the possibility cannot be denied that hepatic branch vagotomy may suppress liver regeneration as a result of alterations of insulin level in the portal vein. However, hepatic branch vagotomy did not cause any significant difference in the serum insulin concentration 7 days after hepatectomy compared with levels in the controls (Figure 5).

It is also likely that liver regeneration may be suppressed by alteration of the blood flow into the liver[20] by section of autonomic nerves. However, in rats subjected to portacaval transposition in order to increase the liver blood flow, tritiated thymidine was incorporated into liver DNA much as it was in the control rats[21]. Section of sympathetic nerves which result in increased blood flow in the liver[22] did not affect liver regeneration (Table 1). In examination of acute changes, section of the hepatic branch of the splanchnic or vagus nerve was noted not to change the blood flow in the hepatic artery or the portal vein. Alterations in blood supply to the liver associated with nerve section play an insignificant role in liver regeneration.

The number of mitotic hepatocytes 3 days after surgery was signficantly lower in the vagotomized group than in the controls, although the value was higher postoperatively than preoperatively (Figure 6). This result supports previous reports that vagotomy delayed and suppressed DNA synthesis[2], this finding is based on the observation that DNA synthesis and mitosis began 15 and 22 h respectively after partial hepatectomy and reached maximum values within the next 10 h[23]. Since such an effect of hepatic branch vagotomy was noted even 7 days after surgery (Figure 6), the effect of denervation appears not to be as short as has previously been reported[2]. However, this could mean that there is a suppression of regenerative response but that it is merely a delay and not a true suppression. Elucidation of its mechanism awaits further studies.

From these observations, we reached the conclusion that the vagus nerve stimulates liver regeneration, and its effect depends on vagal factors directly and specifically.

### *References*

1. Lamar, C., Jr. and Holloway, L.S., Jr. (1977) The effect of vagotomy on hepatic regeneration in rats. *Acta Hepato-Gastroenterology*, **24**, 7–10
2. Kato, H. and Shimazu, T. (1983) Effect of autonomic denervation on DNA synthesis during liver regeneration after partial hepatectomy. *European Journal of Biochemistry*, **134**, 473–478

3. Kraly, F.S., Jerome, C. and Smith G.P. (1986) Specific postoperative syndromes after total and selective vagotomies in the rat. *Appetite*, **7**, 1–17
4. Sakaguchi, T., Yamazaki, M., Tamaki, M. and Niijima, A. (1985) Changes in food intake after hepatic vagotomy at a stage of development in rats. *Neuroscience Letters*, **61**, 317–320
5. Sakaguchi, T. and Yamazaki, M. (1986) Changes in water intake following hepatic vagotomy in young rats. *Journal of the Autonomic Nervous System*, **17**, 243–246
6. Ohtake, M. and Sakaguchi, T. (1987) Alterations in gastric acid secretion following hepatic vagotomy at a stage of development in rats. *International Journal of the Developmental Neuroscience*, **5**, 289–293
7. Stirling, G.A., Laughlin, J. and Washington, S.L.A. (1973) The effects of starvation on the proliferative response after partial hepatectomy. *Experimental and Molecular Pathology*, **19**, 44–52
8. McGowan, J., Atryzek, V. and Fausto, N. (1979) Effects of protein-deprivation on the regeneration of rat liver after partial hepatectomy. *Biochemical Journal*, **180**, 25–35
9. Higgins, G.M. and Anderson, R.M. (1931) Experimental pathology of the liver.I. Restoration of the liver of the white rat following partial surgical removal. *Archives of Pathology*, **12**, 186–202
10. Souto, M. and Echave Llanos, J.M. (1985) The circadian optimal time for hepatectomy in the study of liver regeneration. *Chronobiology International*, **2**, 169–175
11. Ashrif, S., Gillespie, J.S. and Pollock, D. (1974) The effects of drugs or denervation on thymidine uptake into rat regenerating liver. *European Journal of Pharmacology*, **29**, 324–327
12. Sakaguchi, T. and Iwanaga, M. (1982) Effects of D-glucose anomers on afferent discharge in the hepatic vagus nerve. *Experientia*, **38**, 475–476
13. Niijima, A. (1969) Afferent discharges from osmoreceptors in the liver of the guinea pig. *Science*, **166**, 1519–1520
14. Reilly, F.D., McCuskey, P.A. and McCuskey, R.S. (1978) Intrahepatic distribution of nerves in the rat. *Anatomical Record*, **191**, 55–68
15. Madrid, J.A., Salido, G.M. and Martinez de Victoria, E. (1988) Effect of the antimuscarinic agent pirenzepine on the *in vivo* biliary secretion of dogs in response to various stimuli. *Physiologia Bohemoslovaca*, **37**, 67–77
16. Kaplan, M.M. and Righetti, A. (1970) Induction of rat liver alkaline phosphatase: the mechanism of the serum elevation in bile duct obstruction. *Journal of Clinical Investigation*, **49**, 508–516
17. Leffert, H.L., Koch,K.S., Moran, T. and Rubalcava, B. (1979) Hormonal control of rat liver regeneration. *Gastroenterology*, **76**, 1470–1482
18. Goldfine, I.D. (1977) Does insulin need a second messenger? *Diabetes*, **26**, 148–155
19. Yamazaki, M. and Sakaguchi, T. (1989) Pancreatic vagal functional distribution in the secretion of insulin evoked by portal infusion of D-glucose. *Brain Research*, **484**, 357–360
20. Weinbren, K. (1955) The portal blood supply and regeneration of the rat liver. *British Journal of Experimental Pathology*, **36**, 583–591
21. Blumgart, L.H. (1978) Liver atrophy, hypertrophy and regenerative hyperplasia in the rat: the relevance of blood flow. In *Ciba Foundation Symposium 55 (new series): Hepatotrophic factors*, edited by R. Porter and J. Whelan, pp. 181–215. Amsterdam, Elsevier Excerpta Medica.
22. Ackroyd, F.W., Mito, M. and McDermott, W.V., Jr. (1966) Autonomic vasomotor controls in hepatic blood flow. *American Journal of Surgery*, **112**, 356–362
23. Fabrikant, J.I. (1968) The kinetics of cellular proliferation in regenerating liver. *Journal of Cell Biology*, **36**, 551–565

(*Accepted by S. Bengmark 10 September 1992*)

